# Cerebrospinal fluid pressure dynamics reveal signs of effective spinal canal narrowing in ambiguous spine conditions

**DOI:** 10.3389/fneur.2022.951018

**Published:** 2022-08-09

**Authors:** Najmeh Kheram, Nikolai Pfender, Andrea Boraschi, Mazda Farshad, Vartan Kurtcuoglu, Armin Curt, Martin Schubert, Carl M. Zipser

**Affiliations:** ^1^Spinal Cord Injury Center, Balgrist University Hospital, Zurich, Switzerland; ^2^University Spine Center, Balgrist University Hospital, Zurich, Switzerland; ^3^Institute of Physiology, University of Zurich, Zurich, Switzerland

**Keywords:** degenerative cervical myelopathy, spinal cord compression, cerebrospinal fluid pressure, craniospinal compliance, bedside diagnostics, spinal canal, compression biomarker, spine surgery

## Abstract

Spinal canal narrowing with consecutive spinal cord compression is considered a key mechanism in degenerative cervical myelopathy (DCM). DCM is a common spine condition associated with progressive neurological disability, and timely decompressive surgery is recommended. However, the clinical and radiological diagnostic workup is often ambiguous, challenging confident proactive treatment recommendations. Cerebrospinal fluid pressure dynamics (CSFP) are altered by spinal canal narrowing. Therefore, we aim to explore the potential value of bedside CSFP assessments for qualitative and quantitative assessment of spinal canal narrowing in DCM. In this prospective case series, seven patients with DCM underwent bedside lumbar puncture with measurement of CSFP dynamics and routine CSF analysis (NCT02170155). The patients were enrolled when standard diagnostic algorithms did not permit a clear treatment decision. Measurements include baseline CSFP, cardiac-driven CSFP peak-to-trough amplitude (CSFPp), and the Queckenstedt's test (firm pressure on jugular veins) in neutral and reclined head position. From the Queckenstedt's test, proxies for craniospinal elastance (i.e., relative pulse pressure coefficient; RPPC-Q) were calculated analogously to infusion testing. CSFP metrics were deemed suspicious of canal narrowing when numbers were lower than the minimum value from a previously tested elderly spine-healthy cohort (*N* = 14). Mean age was 56 ± 13 years (range, 38–75; 2F); symptom severity was mostly mild to moderate (mean mJOA, 13.5 ± 2.6; range, 9–17). All the patients showed some extent of cervical stenosis in the MRI of unclear significance (5/7 following decompressive cervical spine surgery with an adjacent level or residual stenosis). Baseline CSFP was normal except for one patient (range, 4.7–17.4 mmHg). Normal values were found for CSFPp (0.4–1.3 mmHg) and the Queckenstedt's test in normal head positioning (9.-25.3 mmHg). During reclination, the Queckenstedt's test significantly decreased in one, and CSFPp in another case (>50% compared to normal position). RPPC-Q (0.07–0.19) aligned with lower values from spine-healthy (0.10–0.44). Routine CSF examinations showed mild total protein elevation (mean, 522 ± 108 mg/ml) without further evidence for the disturbed blood brain barrier. Intrathecal CSFP measurements allow discerning disturbed from normal CSFP dynamics in this population. Prospective longitudinal studies should further evaluate the diagnostic utility of CSFP assessments in DCM.

## Introduction

Cerebrospinal fluid (CSF) surrounds the brain and the spinal cord in vertebrates and has numerous functions in the central nervous system (CNS), e.g., buoyancy and homeostasis (1). The CSF space is a dynamic pressure system maintained by finely regulated CSF secretion and absorption (2). CSF pressure dynamics (CSFP) communicate between the cranial and spinal CSF compartments under physiological conditions (3). In presence of cervical spinal canal obstruction, the cranial and spinal pressure compartments become dissociated, and induced elevation of intracranial pressure does not translate to the lumbar level (4). Manual compression of jugular veins with parallel evaluation of lumbar CSFP (the Queckenstedt's test) was routinely done to determine spinal obstruction with moderate to low test sensitivity before it was replaced by spinal neuroimaging (4–7). However, cervical spinal canal stenosis is highly prevalent on MRI, up to 24% in elderly patients (8), and not necessarily associated with clinical symptoms (9). Degenerative cervical myelopathy (DCM) is the umbrella term for patients who develop signs and symptoms from chronic spinal cord compression (10). Dynamic mechanical compression is considered one of the main pathomechanisms in DCM (10). In addition, hyperextension spinal cord injury in preexisting degenerative stenosis is common and attributable to buckled ligamentum flavum and increased disc herniation (11–13). Therefore, the investigation of CSFP dynamics in head reclination is of interest. In patients presenting with clinical evidence of DCM, MRI allows to support the diagnosis and exclude differential diagnoses (14). Generally, timely surgical spinal canal decompression is recommended to halt the disease progression and provide symptom relief (14–16). Surgical decompression is recommended in patients with clinical signs of cervical myelopathy and moderate-severe symptoms of DCM, accompanied by spinal canal narrowing with cord compression (17). In some cases, treatment decision is a challenge. Therefore, evaluating the significance of spinal canal stenosis and biomarkers for predicting the disease course of DCM is required. For this purpose, we aim at exploring the utility of CSFP assessments in ambiguous cases of DCM. Previously, we have provided evidence that intraoperative CSFP dynamics are responsive to surgical decompression (18). For bedside testing, we hypothesized that CSFP dynamics would allow to determine effective spinal obstruction in these ambiguous DCM cases.

## Materials and methods

### Study setting and diagnosis of degenerative cervical myelopathy

The patients with DCM were consecutively enrolled between 2021 and 2022 from the Spinal Cord Injury Center and Department of Neurology and Neurophysiology at Balgrist (NCT02170155) (19). Since diagnostic criteria for DCM are still under development (20), DCM was suspected in patients with clinical signs and symptoms that could be related to cervical myelopathy and spinal canal narrowing on cervical MRI (21). The study protocol conformed to the latest revision of the Declaration of Helsinki and was approved by the local Ethics Committee of the University Hospital of Zurich (KEK-ZH No. PB-2016-00623).

### Study recruitment and clinical examinations

The patients with DCM were included when existing treatment algorithms did not provide a clear recommendation for surgical decompression (10, 17). Standard clinical scores that reflect DCM severity were obtained, i.e., modified Japanese Orthopedic Association (mJOA) and Nurick scores. DCM was graded as mild (defined as an mJOA score of 15–17), moderate (mJOA 12–14), or severe (mJOA < 12).

### CSFP recordings

**Figure 1:**
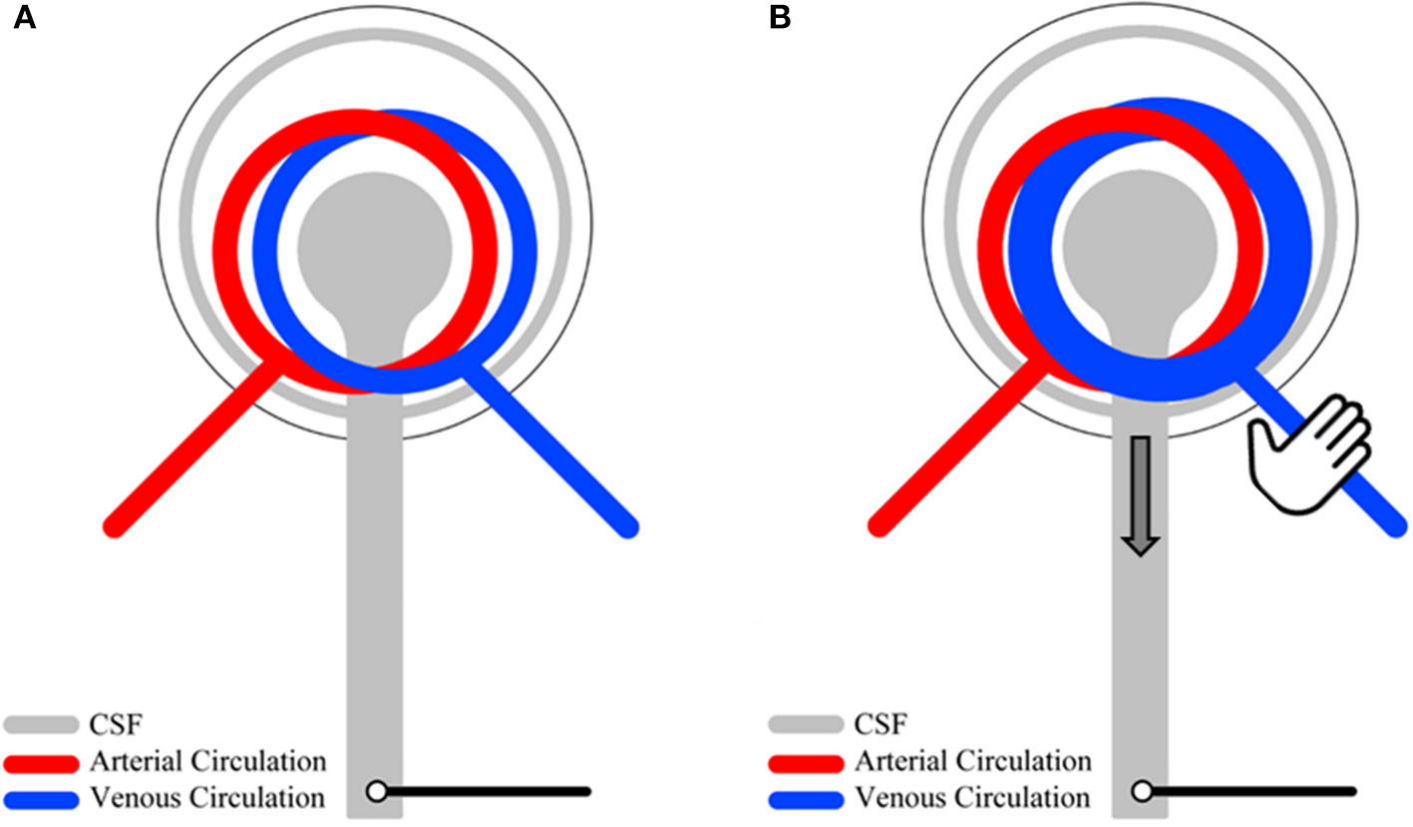
An overview of CSF pressure (CSFP) dynamics. Under physiological resting-state conditions, lumbar cardiac-driven CSFP peak-to-trough amplitude (CSFPp) is a function of intracranial venous filling and arteriolar pulsations **(A)**. During the Queckenstedt's test, intracranial venous filling is temporally increased due to reduced venous outflow, leading to an increased baseline CSFP and increased CSFPp **(B)**.

The patients underwent bedside lumbar puncture (LP) and assessment of CSFP dynamics. LP was done in lateral position with the 20–22 Gauge Sprotte^®^ needles. The needle was connected to an analog to a digital pressure converter (NeuromedexVentrEX), and the digitized signal was linked to a Philips X2-Pat Interface+MX 700 Monitor, connected to online recording software ICM+ (University of Cambridge). The assessment of CSFP dynamics included recording of 30–60 s time windows during resting-state, manual jugular vein compression (the Queckenstedt's test) in neutral head position and during passive head reclination when feasible. A schematic overview of resting-state physiology and mechanisms involved in the Queckenstedt's test is provided (Figure 1).

### CSFP analysis

Data were analyzed with MATLAB. To extract mean CSFP and cardiac-driven CSFP peak-to-trough amplitude (CSFPp), the signal was decomposed into different frequency bins using discrete wavelet decomposition. The sum of the first four frequency bins (0-0.5 Hz) reconstructed mean CSFP, and the sum of the next four frequency bins (0.5–8 Hz) was used for the calculation of CSFPp, which correspond to the difference between the systolic peak and the associated diastolic trough. For the extraction of the relative pulse pressure coefficient, computed from the Queckenstedt's test (RPPC-Q), a regression line was fitted to the CSFPp vs. mean the CSFP curve (the pulsatility curve) formed by individual subjects' data points that were extracted from CSFP data using wavelet decomposition, the data recorded during both the resting state and the Queckenstedt's test. RPPC-Q is the slope of this regression line. CSFP parameters were quantified and compared to those from 14 elderly patients without spinal cord compression previously acquired.

### Ranges of CSFP parameters in patients without spinal cord compression

In general, the comparison to previously acquired ranges for CSFP parameters is limited, as most studies have not calibrated their measurement system with respect to geometric and elastic properties of the measurement components (e.g., the lumbar needle itself and connecting tubes). The range of CSFP rise from the baseline during the Queckenstedt's test is highly variable, and a normal range has not been defined. In historical studies, clinical judgment mainly depended on the presence or absence of CSFP response. Prior to this study, we acquired CSFP data with the same technical setup from 14 elderly patients who underwent lumbar puncture for reasons other than spinal cord compression. Mean age was 59.7 ± 9.3 years (39–73), 6F, mean BMI was 25 ± 3 (18–30). All patients were in stable medical condition (outpatient setting), and there was no evidence for stenosis of the cervical spinal canal. Lumbar puncture was done for suspicion of demyelinating disease in most patients (*N* = 7), and for peripheral neuropathy or infectious CNS disease in the remaining patients. In that cohort, median CSFPp was 1. mmHg [the interquartile range, (IQR) 0.5], ranging from 0.4 to 2.1 mmHg. The CSFP rise during the Queckenstedt's test was 12.5 mmHg [7.3]; range, 5.3–34.1 mmHg. Median RPPC-Q was 0.18 [0.04]; range, 0.1–0.4. For comparison, we followed a conservative approach, setting the cut-off to values lower than the lowest numbers found in the control cohort, i.e., <8.2 mmHg for baseline CSFP, <0.4 mmHg for CSFPp, <5 mmHg for the maximum CSFP rise during the Queckenstedt's test, and <0.1 for RPPC-Q.

## Results

### Patient characteristics

**Figure 2:**
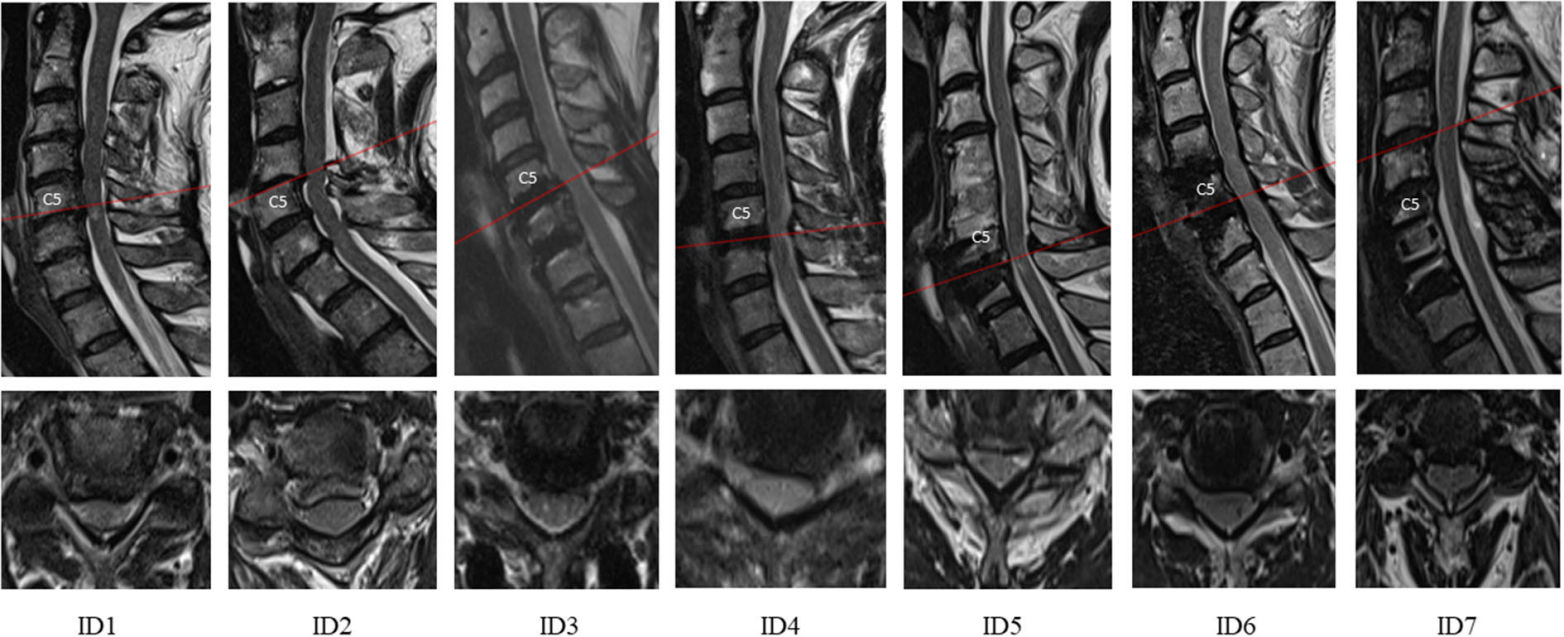
Sagittal and axial cervical MRI from the time of consultation shown for all subjects (ID1-7). The red line indicates the level of maximum stenosis. Spine imaging shows that all patients had spinal canal stenosis; T2-hyperintensity was present in 5 out of 7. Structural changes from previous spine surgery can be seen (ID3-7, e.g., anterior decompression with fusion in ID7).

Seven patients were enrolled (mean age, 56 ± 13 years; range, 24–75 years, 2F; BMI, 26 ± 7; range, 19–37). Mean age and BMI of patients with DCM were not different from the spine-healthy cohort (*p* = 0.494 and *p* = 0.829, respectively). DCM was mostly mild or moderate and severe in one case (mean mJOA, 13.5 ± 2.6; range, 9–17). All the patients tolerated LP and CSFP assessments well without adverse events. Most patients have undergone decompressive surgery previously (5/7) and had residual stable or progressive symptoms and adjacent level stenosis or residual at-level stenosis of unclear significance. The remaining patients (2/7) had very mild DCM and ambiguous stenosis of unclear significance. The mean cross-sectional area (CSA) of spinal canal was 99 ± 11 mm^2^, and mean CSF-CSA was 39 ± 15 mm^2^. Individual clinical characteristics and detailed CSFP findings are summarized in Table 1. The patients' cervical MRIs are shown in Figure 2.

**Table 1:** Clinical characteristics and detailed CSFP findings of patients with degenerative cervical myelopathy (DCM).

**ID**	**Type of** **disease**	**mJOA/** **Nurick**	**Time since** **onset**	**Max. level of** **stenosis, no.** **stenotic levels** **(+/- myelopathy)**	**CSF-CSA/CSA** spinal canal*	**RS** **CSFP†/††/§**	**RS** **CSFPp†/††/§**	**CSFP rise** **due to** **Queckenstedt's** **test†/§**	**RPPC-** **Q^§^**
Range from spine-healthy	NA	NA	NA	NA	NA	8.6–18.9	0.4–2.2	5.3–34.1	0.10–0.44
1	DCM	15/2	>10 yr.	C5, 4 (+)	29.5/83.6	10.4 [0.4]	0.4 [0.3]	15.9	0.12
2	DCM	17/0	<1 yr.	C4, 3 (-)	24.7/101.5	4.7 [1.0]|4.3 [0.7]	0.4 [0.2]|0.4 [0.3]	15.0|7.2	0.12 |NA
3	DCM, post-surgery	15/1	2–3 yr.	C5, 1 (+)	49.9/112.6	13.0 [0.4]	0.8 [0.3]	25.3	0.19
4	DCM, post-surgery	13/2	2 yr.	C5, 1 (+)	32.3/90.8	13.5 [0.2]|10.5 [0.4]	0.7 [0.2]|0.3 [0.3]	9.0|10.5	0.07|0.07
5	DCM, post-surgery	12/3	2 yr.	C6, 2 (+)	55.9/98.5	10.1 [0.3]|14.1 [0.8]	1.3 [0.2]|1.3 [0.5]	21.3|27.4	0.10|0.06
6	DCM, post-surgery	14/2	4 yr.	C4, 2 (+)	23.7/95	16.0 [0.8]|14.8 [1.1]	0.8 [0.3]| 0.6 [0.3]	10.6|10.2	0.19|0.13
7	DCM, post-surgery	9/5	6 yr.	C3, 1 (+)	57.2/112.8	17.4 [0.5] | 18.5 [0.7]	0.5 [0.1]|0.4 [0.1]	17.7|18.7	0.09|0.14

^*^mm2 at max. stenosis † in mmHg; †† median, [interquartile range]; § during neutral head position | head reclination; AIS, ASIA Impairment Scale; CSFP, Cerebrospinal Fluid Pressure; CSFPp, Cardiac-driven CSFP peak-to-trough amplitude; DCM, Degenerative Cervical Myelopathy; mJOA, modified Japanese Orthopedic Association scale; NA, not applicable; RPPC-Q, Relative Pulse Pressure Coefficient acquired through Queckenstedt's test; RS, resting-state.

### CSF examinations

Total CSF protein was available from all patients (522 ± 108 mg/ml). Compared to the lab-specific ranges (200–500 mg), it was mildly elevated in four patients (518, 541, 670, and 600 mg; ID1-3 and 5). The remaining CSF examinations were within normal ranges. Lactate was 2 ± 0.3 mmol/l (lab-specific range, 1.7–2.6 mmol/l). From *N* = 6, the CSF cell count (mean, 1.3 ± 0.5/microliter; lab-specific range, 0–4/microliter) and glucose (3.6 mmol/l; lab-specific range, 2.7–4.2 mmol/l) were collected. Oligoclonal bands and Reiber analytics were done in *N* = 3 (ID1-3); no abnormalities were found.

### CSFP metrics and the calculation of RPPC-Q

**Figure 3:**
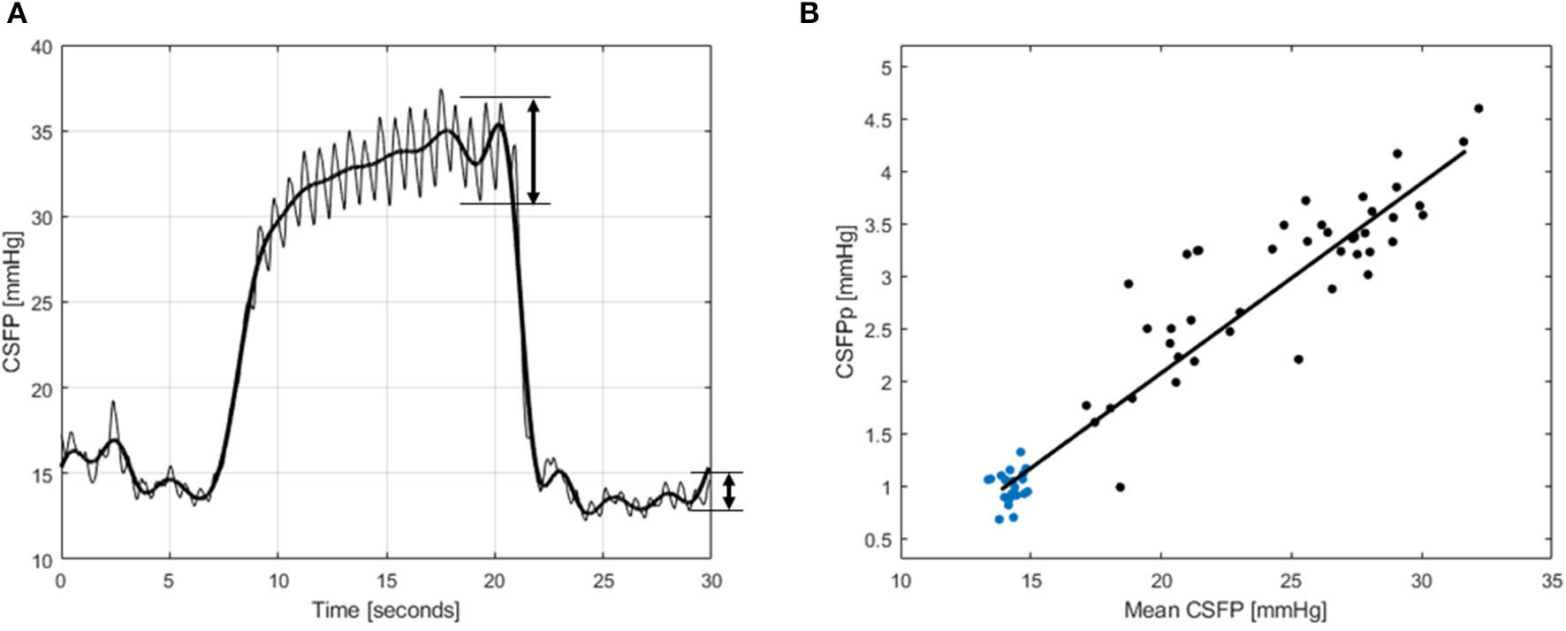
A schematized illustration for the CSF pressure (CSFP) metrics from a spine-healthy patient. In **(A)**, CSFP is shown, the bold line is mean CSFP, and the cardiac-driven CSFP amplitude is shown at the end of the Queckenstedt's maneuver (20 s) and at the end of the recording, where CSFPp is calculated by subtraction of peak values from the previous trough. In **(B)**, the pulsatility curve is shown for the same CSFP recording, where the blue points are values from the steady state and the black points are from the Queckenstedt's test. The slope of the line fitted to the points is RPPC-Q.

Figure 3 illustrates a representative CSFP measurement during the Queckenstedt's test in a spine-healthy patient from a previously tested cohort. In Figure 3A, the black line represents CSFP recording, the red line is mean CSFP (0 −0.5 Hz), and the blue line is cardiac-driven CSFP amplitude (0.5–8 Hz), where CSFPp can be calculated by subtraction of the peak values from the previous trough. Each pair of mean CSFP and CSFPp from the steady state (blue points) and during the Queckenstedt's test (black points) is plotted on the pulsatility curve (Figure 3B), and the slope of the fitted regression line is equal to RPPC-Q.

### Group of patients that did not previously undergo surgery

ID1: In this patient, DCM was diagnosed for over a decade with mild stable spasticity, gait ataxia, and dexterity impairment, while imaging showed multilevel spinal canal stenosis and T2-weighted hyperintensity at level C5-7. CSFP findings were borderline for median CSFPp (0.4 mmHg), but, otherwise, within ranges acquired from the patients without spinal cord compression.

**Figure 4:**
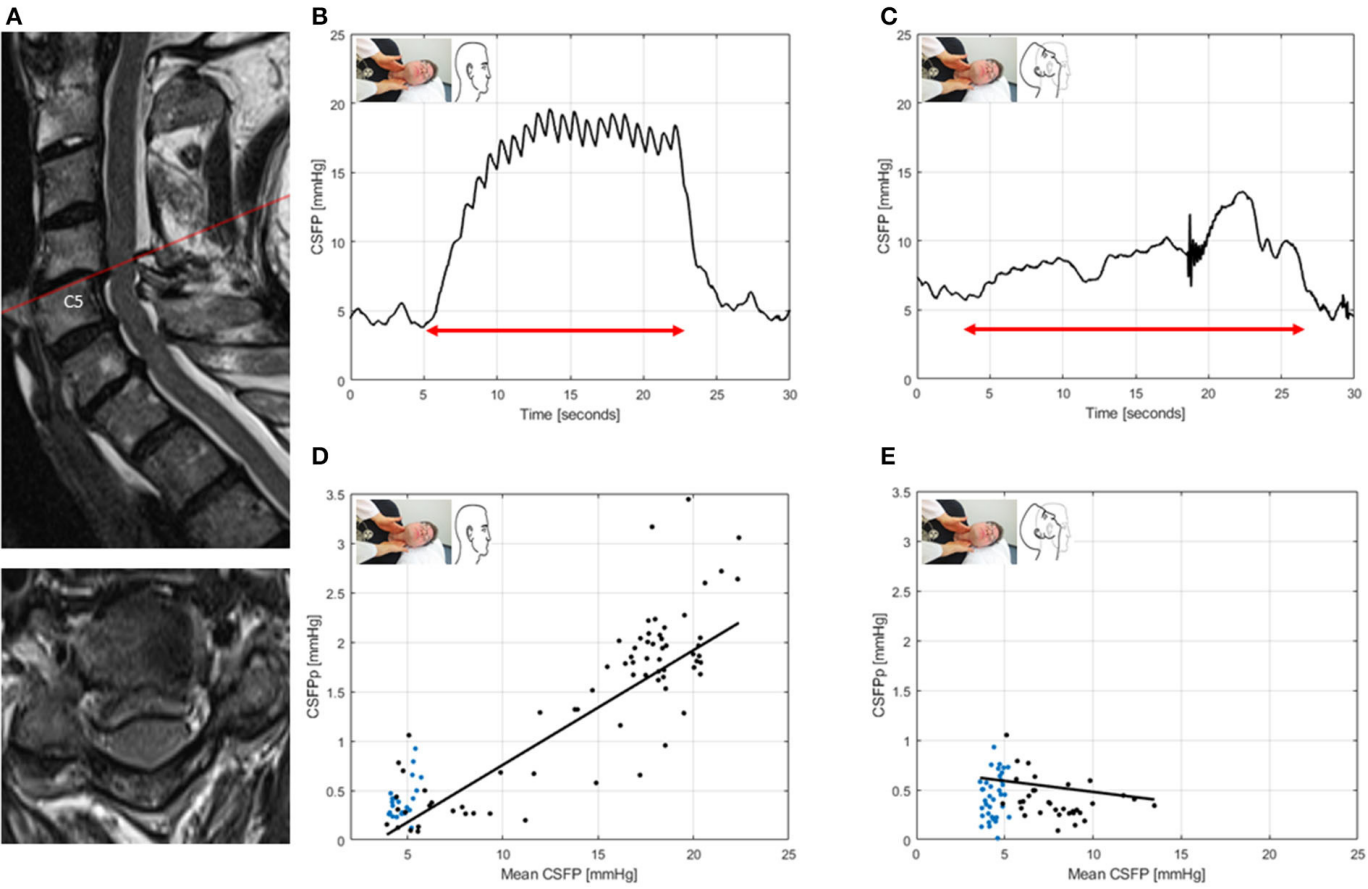
T2-weighted MRI of the cervical spine for ID2 (patient with degenerative cervical myelopathy, mJOA: 17) **(A)**. Sagittal and axial images showing spinal canal stenosis maximum at level C4/5, without clear cord compression. CSFP is shown during the Queckenstedt's test in neutral head position (rise, 15.2 mmHg) **(B)**, which declines in head reclination (rise, 7.2 mmHg) **(C)**. Cardiac-driven CSFP peak-to-trough amplitude (CSFPp) at resting state (blue dots) and during the Queckenstedt's test (black dots) is plotted against mean CSFP **(D)**. The regression line (in black) is reported as relative pulse pressure coefficient (RPPC-Q). In neutral head position, RPPC-Q is 0.12 **(D)**, and, in head reclination, it declines to −0.02 **(E)**.

ID2: This patient had mild DCM and spinal canal stenosis without significant cord compression. In neutral head position, baseline CSFP was low (4.7 mmHg), the CSFPp borderline (median, 0.4 mmHg), and Queckenstedt's rise normal (15. mmHg). During head reclination, CSFPp did not change, while the Queckenstedt's test revealed a partial block (Figure 4).

### Group of patients that previously underwent surgery

ID3: This patient underwent anterior decompressive surgery C5/6 and C6/7 and dorsal instrumentation C6-T1 with laminectomy C7. The patient presented with residual neuropathic pain in the left C6 dermatome with residual mild cord compression on MRI. CSFP dynamics were within ranges acquired from the spine-healthy comparison group. Head reclination was not possible due to neck pain associated with the maneuver.

**Figure 5:**
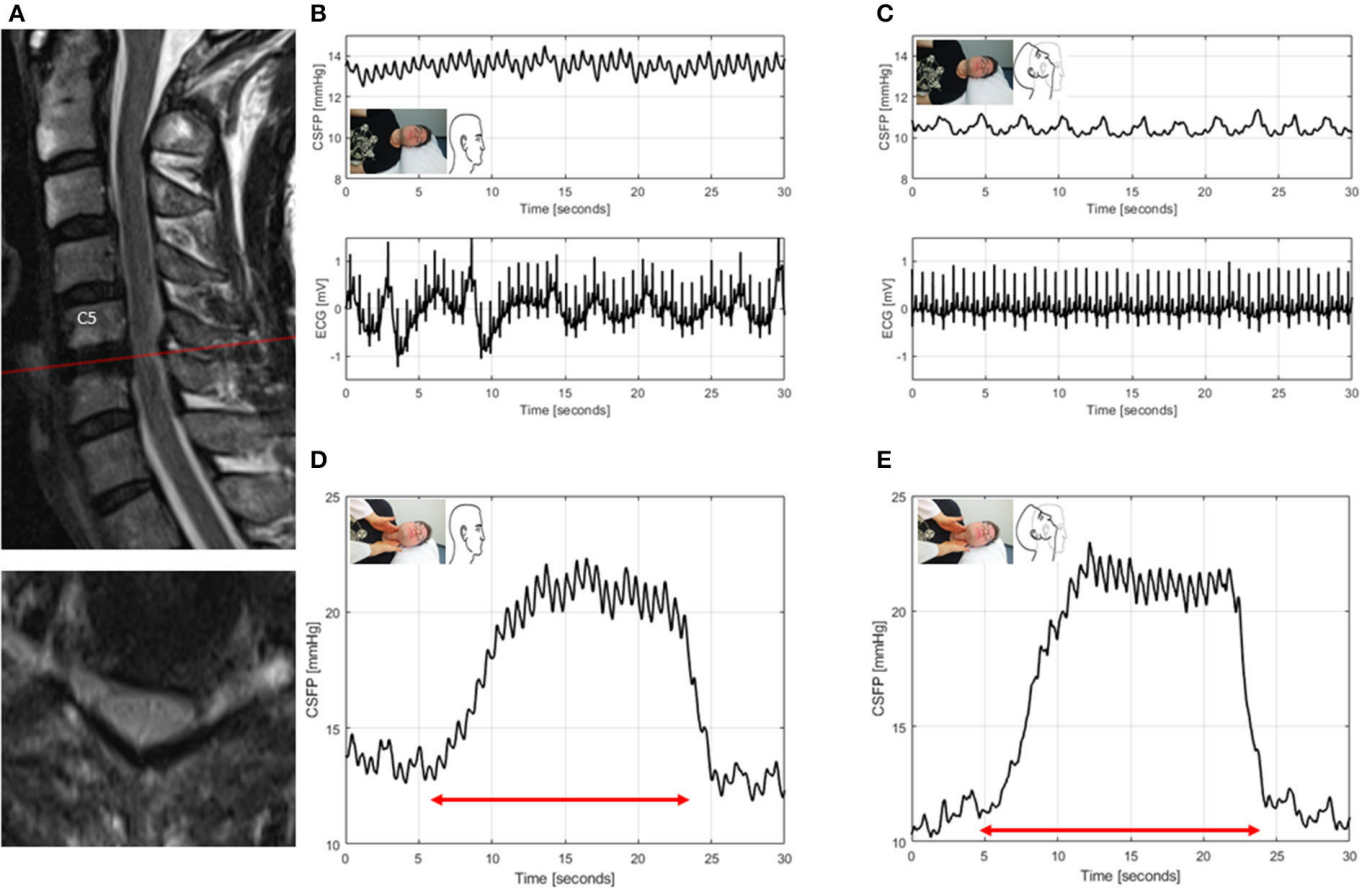
T2-weighted MRI of the cervical spine for ID4 (patient with spinal canal stenosis of unknown significance, previously underwent surgery for degenerative cervical myelopathy, mJOA: 13) **(A)**. Sagittal and axial images show posterior cord compression at levels C5/6. CSFP and electrocardiogram (ECG) are shown during resting state in neutral head position with baseline CSFP of 13.5 mmHg and cardiac-driven CSFP peak-to-trough amplitude (CSFPp) of 0.7 mmHg **(B)**, and, in head reclination, baseline CSFP and CSFPp both declined to 10.5 mmHg and 0.3 mmHg, respectively **(C)**. CSFP during the Queckenstedt's test in neutral head position (rise, 9. mmHg) **(D)**, and, in head reclination, (rise, 10.5 mmHg) **(E)**.

ID4: This patient underwent anterior decompressive surgery with fusion C5/6 (ACDF). The spastic gait disorder, which was present before surgery, did not improve following decompression. MRI revealed residual posterior cord compression with unclear clinical significance. CSFP dynamics showed normal median CSFPp (0.7 mmHg), and the Queckenstedt's rise was 9. mmHg in neutral head position, whereas CSFPp decreased during head reclination (0.7 vs. 0.3 mmHg). The CSFP rise due to the Queckenstedt's test did not change during head reclination (Figure 5).

ID5: This patient has previously undergone revision ACDF C6/7 due to adjacent level stenosis related to previous decompression surgery C4/5 and C5/6. This patient improved in spastic gait disorder after decompression but developed gait worsening 5 months post-surgery. MRI of the cervical spine showed no change in the myelopathy signal and a constant moderate posterior spinal canal narrowing. Despite an optimized metal artifact reduction sequence (MARS) (22), it was not possible to exclude residual (posterior) effective cord compression. Regarding CSFP dynamics, compared to neutral head position, RPPC-Q was slightly reduced during head reclination (0.1 vs. 0.06).

ID6: The patient has previously undergone anterior decompressive surgery C4-C6 with disc replacement and had residual neuropathic pain in C7 dermatome, mild dexterity dysfunction, and ataxia and was referred to our department as MRI showed cervical spinal canal stenosis of unclear clinical significance. CSFP dynamics were within ranges acquired from spine-healthy patients.

**Figure 6:**
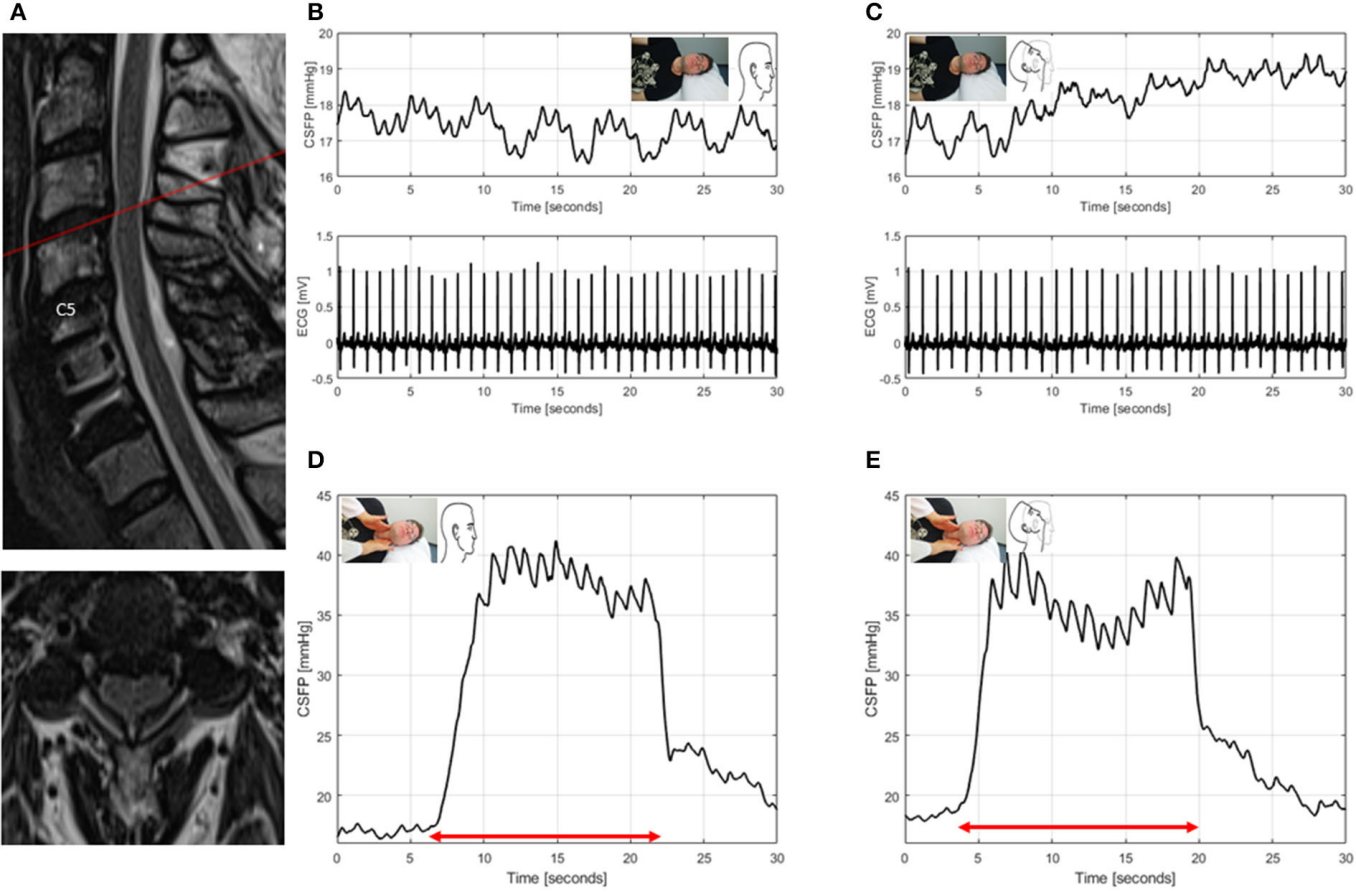
T2-weighted MRI of the cervical spine for ID7 (patient with spinal canal stenosis of unknown significance, previously underwent surgery for degenerative cervical myelopathy, mJOA:9) **(A)**. Sagittal and axial images showing spinal canal narrowing with spinal cord induration at level C3/4. CSFP and electrocardiogram (ECG) are shown during resting state in neutral head position with baseline CSFP of 17.4 mmHg and cardiac-driven CSFP peak-to-trough amplitude (CSFPp) of 0.5 mmHg **(B)**, and in head reclination with baseline CSFP of 18.5 mmHg and CSFPp of 0.4 mmHg **(C)**. CSFP is plotted during the Queckenstedt's test in neutral head position (rise, 17.7 mmHg) **(D)**, and in head reclination (rise, 18.7 mmHg) **(E)**.

ID7: The patient has previously undergone ACDF and open door laminoplasty C5/6 and had chronic neck pain and incomplete tetraplegia. Wheelchair-dependence was evident from the first surgery on, with currently progressive sensory deficits of the arms and the legs. MRI showed adjacent level stenosis at C3/4, which was initially present after the first surgery and remained unchanged ever since. Regarding CSFP dynamics, only RPPC-Q was borderline in neutral head position and normal in head reclination (0.09 vs. 0.14) (Figure 6).

## Discussion

### Summary of main findings

Treatment decision for DCM was not clear in patients that had residual symptoms following surgery, or mild DCM with ambiguous spinal canal stenosis. This study investigated CSFP dynamics to determine effective cord compression in these patients during normal head position and during dynamic canal narrowing through head reclination. During head reclination, but not in normal head position, there were abnormal CSFP dynamics in one patient with residual symptoms following surgery and one surgery-naïve patient with ambiguous stenosis. Specifically, there was a partial block in the Queckenstedt's test in one, and decrease of CSFP pulsations in the other patient. In the remaining patients, despite spinal canal stenosis, CSFP dynamics were not different from a spine-healthy comparison group in both positions. These findings suggest that normal and abnormal CSFP dynamics can be discerned, and that dynamic head positioning increases the sensitivity to detect abnormal CSFP dynamics in DCM. As an additional finding, there were no severe abnormalities in CSF analysis.

### Current treatment algorithms in DCM

Current clinical practice guidelines strongly recommend decompressive surgery in newly diagnosed patients with moderate-severe DCM, and weakly recommend surgery in patients with neurological deterioration (17). Presumably, patients benefit most from an early intervention when symptoms have higher chances of being reversible. However, there are several reasons for uncertainty in these patients. From a methodological point of view, the recommendations were not stronger due to imprecise and varying definitions of DCM in the literature (20). This led to the mixing up of patients with DCM and those presenting with asymptomatic spine stenosis or radiculopathy. In clinical practice, however, spinal cord compression, as revealed in cervical MRI, remains a key factor in surgical decision-making (23). More importantly, comprehensive decision-making relies on weighing the risks and benefits of surgery and alternative strategies (14). Thus, the risk of neurological deterioration in patients with DCM, which is presumably high as recently demonstrated in a retrospective cohort study (24), must be considered against surgical complications (e.g., C5 palsy) and unfavorable long-term sequelae (e.g., adjacent level stenosis) (25). In two large cohort studies in patients with mild DCM (15, 26), there was supporting evidence of a significant functional benefit from surgery at 3- to 24-month follow-up. One of these studies reported adverse events at 3-month follow-up in 27.7% of patients (26). Of interest are efforts to improve prediction of the disease course in patients with mild DCM based on machine learning algorithms and specific clinical constellations (27, 28). Notably, current guidelines do not cover the many cases of patients with residual symptoms who already underwent surgical decompression, where it is a commonly encountered challenge to discern those with residual effective cord compression and those who did not recover following surgery.

### Interrelations between CSFP dynamics and neuroimaging in DCM

The pathophysiology of DCM is still incompletely understood, and its investigation was defined a key research priority in DCM (29). Imaging is a major asset in the evaluation of cord distress, providing structural information as well as dynamic parameters, such as CSF flow and cord motion (30). It has resulted in an abundance of features, which consequently introduced challenges of ambiguity and comparison (31, 32). In our cohort, we had cervical MRI to visually evaluate the degree of stenosis, which failed to align with CSFP dynamics in all cases, with the former frequently suggesting higher severity. There are several explanations for this finding. Standard MRI is not directly linked to CSFP, since it does not provide sufficient temporal resolution to detect changing geometry of the CSF space during a cardiac cycle; hence, it does not contain any quantitative information about its elastic properties. Similarly, changes induced through head reclination are not considered. Lastly, standard MRI may be insensitive to preserved space in the spinal canal where CSF can pass. Thus, advanced imaging methods better suited to reflect the functional properties of the CSF pressure system are required.

### CSFP dynamics in DCM: Methodological considerations

In this cohort, all patients had some degree of spinal stenosis and symptoms suggestive of residual DCM. Interestingly, structural evidence of spine stenosis from static MRI was not systematically associated with disturbed CSFP dynamics measured at the lumbar level. Assuming that spinal obstruction was affecting cranio-caudal CSF dynamics, this finding suggests that CSFP dynamics provide additional information for the functional relevance of spinal canal stenosis. We consider disturbed CSFP dynamics to potentially reflect spinal cord distress since the cord is presumably more vulnerable to repetitive trauma (i.e., head reclination or neck contusion) when the spinal canal is effectively obstructed. Subsequently, the patients with disturbed CSFP dynamics would have a higher risk for neurological deterioration and to be referred for surgery. In contrast, the patients with unsuspicious CSFP dynamics may be followed up closely and referred for conservative management. From the performed assessments, head reclination was most valuable to reveal CSFP abnormalities. This finding is in line with a study that systematically assessed the Queckenstedt's test during different spine positions in 85 moderately to severely affected patients with DCM and found the highest likelihood of abnormal Queckenstedt's response during head reclination (33). Our findings suggest that the position-dependent spinal block can also be detected in patients with mild myelopathy. Hence, further studies are required to confirm the notion that patients with disturbed CSFP dynamics also have a higher risk for deterioration. Importantly, surgery may still be necessary in patients with mild DCM who show progressive symptoms or in those with modifying cofactors, such as severe pain or medical comorbidities (e.g., increased risk of falls with acute-on-chronic injuries).

### The Queckenstedt's test in DCM: Physiology and comparison to the literature

The Queckenstedt's test is performed by manual compression of the jugular vein, leading to an increase of venous blood volume in the brain and, consequently, the rise of CSFP and CSFPp (34). Previously, we have incorporated this maneuver in a spine-healthy cohort to evaluate CSF space elastance by extracting RPPC-Q as its proxy. Cervical blockage is associated with a hindered communication of the cerebral and spinal CSF space (4), thus limiting the CSF volume shift during each cardiac cycle and hypothetically affecting the proxies for elastance as computed with the Queckenstedt's test; however, this has not been tested in cervical blockage yet. Similarly, the effect of dynamic cord compression on CSFP parameters can be revealed during head reclination (33). The traditional interpretation of the Queckenstedt's test distinguished “complete spinal block” as defined by absence of CSFP response and “partial block” as defined by the block during head movements. It is important to note that our findings of this study cannot be generalized to the whole DCM population due to the high anatomical and clinical variability of this pathology. Additionally, a comparison with previous studies that applied the Queckenstedt's test, among which some included patients with DCM, is challenging due to varying cohort characteristics. While we explored cases of mildly affected patients with DCM who suffer from extra-dural spinal cord compression, most historical studies included patients with suspicion of cord compression without further specification if intra-dural compression was present (35). Regarding the CSFP rise, all the values were found within the previously obtained healthy range. “Spinal block” according to traditional definition (i.e., absence of Queckenstedt's response) was not found in any patient in this cohort, indicating the robustness of this parameter in DCM and that, perhaps, extra-dural spinal cord compression has less of an effect on CSFP dynamics than intra-dural compression.

### The advanced Queckenstedt's test

The RPPC-Q, as determined during the Queckenstedt's test, is considered a metric for investigation of the biomechanical properties of the CSF compartment, analogously to RPPC calculated from infusing testing. In several cases (5/7), RPPC-Q was in the lower quartile of values acquired from the spine-healthy cohort. In ID2, it was observed that, compared to a neutral neck position, head reclination severely hindered the communication between the CSF cranial and spinal compartments. In this case, the Queckenstedt's test was unresponsive during head reclination, i.e., the rise of mean CSFP did not even reach the region of the pulsatility curve where a linear relationship exists between mean CSFP and CSFPp. This translates into the fact that RPPC-Q could not be adequately computed for this patient during head reclination. Due to paucity in the literature about viscoelastic properties of the CSF compartment in DCM and a lack of further studies on RPPC-Q, a proven explanation for these findings cannot be provided. In general, reduced RPPC-Q in DCM may indicate a lack or slowing of filling of a lumbar compartment or changes of elastic properties of the spinal CSF compartment.

### CSFP dynamics in spinal cord diseases: Potential diagnostic applications

Assuming that CSFP assessments enabled the identification of patients with dynamic cord compression in this case series, we consider the systematic longitudinal investigation of CSFP dynamics promising in larger cohorts of patients with ambiguous cord compression. From all CSFP metrics obtained, head position-induced changes appeared to be of highest value, because reclination showed clear effects on CSFP dynamics (best visible in RPPC-Q for resting state and during reclination in ID4 and a lack of the CSFP rise during the Queckenstedt's test in ID2), suggesting that the maneuver thus may serve as a provocation test for functional stenosis, thereby helping to determine functionally relevant canal narrowing. Although advanced neuroimaging does provide valuable information about effective canal obstruction, derived from CSF flow and cord motion data, it offers no insight into the pressure in the CSF compartment and, therefore, cannot substitute for CSFP assessments. In addition, due to artifacts in patients that have already undergone surgery with instrumentation, it is difficult to successfully apply and evaluate advanced imaging sequences. Previous studies have reported abnormal CSF examinations, indicating a disturbed blood brain barrier in moderate to severe DCM (36). Our preliminary CSF analysis findings did not reveal severe abnormalities, and are suggestive that the blood brain barrier is not significantly altered in mild DCM and patients with residual deficits. Beyond diagnostic purposes, CSFP assessment may provide surrogate markers for cord compression in clinical trials. Studies that investigate the efficacy of intrathecal cell-/drug-/stem-cell therapies (30) may easily obtain CSFP dynamics during administration, which provides an additional biomarker to help identify factors relevant for treatment response (i.e., better understanding profiles of treatment responders and non-responders). Since most therapeutic trials were conducted in patients with acute spinal cord injury, and given different injury mechanisms involved in SCI (i.e., swelling and edema) (37) as compared to DCM (i.e., chronic cord compression and ischemia) (38), the investigation of CSFP dynamics in SCI warrants further attention.

### Strengths and limitations

This study was novel for investigating bedside CSFP dynamics in patients with mild DCM or those who have previously undergone surgery. This patient population is of particular interest, as current treatment algorithms do not allow for strong management recommendations. Data from spine-healthy patients were available for comparison, and the measurement system has been tested previously to allow for inter-trial comparability. Despite these strengths, some limitations need to be mentioned. First, this is a small case series that primarily aimed to identify DCM subpopulations that could benefit from CSFP assessments and to obtain pilot data in these patients. Second, the data were gathered from single measurements, which may be affected by the condition of the patient and the measuring system during the time of acquisition. Therefore, averaging multiple acquisitions could provide more robust values. Third, changes of the head position were not available in all patients due to decreased flexibility of the cervical spine after fusion. Fourth, RPPC-Q was not obtainable in all the cases, as in ID2 during head reclination. It is important to note that, even though a supposedly complete spinal block limits the applicability of RPPC-Q, for such cases, the mean CSFP rise was shown to be sensitive. Longitudinal studies are needed to further elaborate on the association between CSFP dynamics and clinical deterioration in DCM. Furthermore, the sensitivity of CSFP dynamics for surgery-related changes warrants a pre- to postoperative testing. Lastly, potential confounders of CSFP dynamics, i.e., arterial blood pressure, body-mass index, or age, were not systematically analyzed, owed to the case-based analysis. This approach was preferred over a group-based approach to account for individual patient characteristics. In addition, factors, such as pain, that may increase intra-abdominal pressure and, consequently, raise central venous pressure must be considered.

## Conclusion

In patients with mild DCM and residuals following decompressive surgery, diagnostic tools for determination of effective cord compression are required. With bedside intrathecal CSFP assessments, it was possible to distinguish disturbed from normal CSFP dynamics. Longitudinal studies, including pre- to post-decompression measurements, are needed to determine the sensitivity of CSFP dynamics for effective cord compression.

## Data availability statement

The original contributions presented in the study are included in the article/supplementary material, further inquiries can be directed to the corresponding author.

## Ethics statement

The studies involving human participants were reviewed and approved by Ethics Committee of the University Hospital of Zurich. The patients/participants provided their written informed consent to participate in this study. Written informed consent was obtained from the individual(s) for the publication of any potentially identifiable images or data included in this article.

## Author contributions

CZ and NK: conception and design of the study, acquisition and analysis of data, data interpretation, and drafting a significant proportion of the manuscript and figures. NP and AB: acquisition and analysis of the data and data interpretation. MF, VK, AC, and MS: data interpretation. All authors contributed to the final manuscript, contributed to the article, and approved the submitted version.

## Funding

CZ, AC, and MS report a grant from the Swiss Paraplegia Foundation (FoKo_2019_01) and VK reports a grant from the Swiss National Science Foundation (Project No. 182683).

## Conflict of interest

The authors declare that the research was conducted in the absence of any commercial or financial relationships that could be construed as a potential conflict of interest.

## Publisher's note

All claims expressed in this article are solely those of the authors and do not necessarily represent those of their affiliated organizations, or those of the publisher, the editors and the reviewers. Any product that may be evaluated in this article, or claim that may be made by its manufacturer, is not guaranteed or endorsed by the publisher.

## Abbreviations

ACDF: Anterior Decompressive Surgery with Fusion
ASIA: American Spinal Injury Association
CSFP: Cerebrospinal fluid pressure
CSFPp: Cardiac-induced CSFP peak-to-trough amplitude
DCM: Degenerative cervical myelopathy
ISNCSCI: International Standards for Neurological Classification of Spinal Cord Injury
LP: Lumbar puncture
mJOA: modified Japanese Orthopedic Association Score
MRI: Magnetic resonance imaging
RPPC-Q: Relative pulse pressure coefficient computed through Queckenstedt's test
SCI: Spinal cord injury.
